# Abdominal surgical site infections: incidence and risk factors at an Iranian teaching hospital

**DOI:** 10.1186/1471-2482-5-2

**Published:** 2005-02-27

**Authors:** Seyd Mansour Razavi, Mohammad Ibrahimpoor, Ahmad Sabouri Kashani, Ali Jafarian

**Affiliations:** 1 Community medicine department, school of medicine, Tehran university of medical sciences, Tehran, Iran; 2 school of medicine, TUMS, Tehran, Iran; 3 Educational Development Center, TUMS, Tehran, Iran; 4 surgery department, school of medicine, TUMS, Tehran, Iran

## Abstract

**Background:**

Abdominal surgical site infections are among the most common complications of inpatient admissions and have serious consequences for outcomes and costs. Different risk factors may be involved, including age, sex, nutrition and immunity, prophylactic antibiotics, operation type and duration, type of shaving, and secondary infections. This study aimed to determine the risk factors affecting abdominal surgical site infections and their incidence at Imam Khomeini, a major referral teaching hospital in Iran.

**Methods:**

Patients (n = 802) who had undergone abdominal surgery were studied and the relationships among variables were analyzed by Student's t and Chi-square tests. The subjects were followed for 30 days and by a 20-item questionnaire. Data were collected through pre- and post-operative examinations and telephone follow-ups.

**Results:**

Of the 802 patients, 139 suffered from SSI (17.4%). In 40.8% of the cases, the wound was dirty infected. The average age for the patients was 46.7 years. The operations were elective in 75.7% of the cases and 24.7% were urgent. The average duration of the operation was 2.24 hours, the average duration of pre-operative hospital stay 4.31 days and the average length of (pre- and post-operation) hospital stay 11.2 days. Three quarters of the cases were shaved 12 hours before the operation. The increased operation time, increased bed stay, electivity of the operation, septicity of the wound, type of incision, the administration of prophylactic antibiotic, type of operation, background disease, and the increased time lapse between shaving and operation all significantly associated with SSI with a p-value less than 0.001.

**Conclusion:**

In view of the high rate of SSI reported here (17.4% compared with the 14% quoted in literature), this study suggests that by reducing the average operation time to less than 2 hours, the average preoperative stay to 4 days and the overall stay to less than 11 days, and approximating the timing of shaving to the operation and substituting cefazolin for cefaluthin when prophylactic antibiotic is to be administered, the SSI may be reduced to a more acceptable level.

## Background

SSIs are among the most common hospital acquired infections comprising 14–16 percent of inpatient infections[1]. SSI is a dangerous condition, a heavy burden on the patient and social health system[2]. Such infections lengthens bed stay for an average of seven days. Potential sources of infection are the patient (especially contamination by alimentary tract bacteria), hospital environment, food, other patients, staff, infected surgical instruments, dressings, and even drugs and injections [3]. The incedence of SSIs with regard to abdominal surgical sites and operating conditions is as follows:

Clean wounds (1.5–3.7%); clean-contaminated wounds (3–4%); contaminated wounds (8.5%); dirty-infected wounds (28–40%); in laparoscopy (10%) umbilical hernia (2–5%); in the cancer of the colon without taking antimicrobial drugs (30–60%); or with antibiotic and proper intestine wash (10%); in colostomy (above 50%); in colon perforation (20%); in stomach cancer and surgery (20%); in hernionite (50%); in adult appendectomy (10–20%); in children's appendicitis (2–5%); in aged appendicitis and in pregnant women (10–50%); and in AIDS victims (above 50%); in liver abscess (20%); in hydatid cyst (2–5%); in acute and chronic cholecystectomy without stones (10%); in acute septic cholangitis (10–20%); in laparoscopic cholecystectomy (2–5%); and in splenectomy (2–5%) [4].

SSI is identified with redness, inflammation, heat, pain, a temperature of 38°C, and septic drainage from the surgical site during the 30 days following operation[4]. Several factors are to be taken into account for SSI, some of which are as follows:

While we could not find any significant correlation between sex and SSI rate, age proved to be an important factor; the rate of wound infection for 15 to 24-year-old patients was only10% but increased significantly for those over 65 years of age. The extent of SSI was doubled for obese patients. The duration of surgical operation also proved to be a significant factor: only 3% of operations lasting 30 minutes or less led to infection, while for operations lasting more than 6 hours this rate leapt to 18%. SSI rate increased with longer durations of preoperative bed stay, but preoperative showers with a disinfecting soap such as chlorhexidine or Betadine decreased the cutaneous bacterial load. According to Kruise, the rate of infection was reduced to 1.3% among patients who showered with disinfecting soap containing hexachlorophene. In those who showered with ordinary soap the corresponding figure was 2.1%, and for patients who did not shower at all it increased to 2.3%. However, another study on 5536 subjects showed no decrease in SSI in patients who showered with chlorhexidine preoperatively; the rate was 4% [4]. Among other factors that delay wound healing or increase the infection rate are cigarette smoking, which increases the postoperative infection rate 5-fold, and the use of steroids, which delays wound healing and increases the infection rate by 9 percentage points from 7% to 16%. Aseptic surgical techniques are claimed to decrease the infection rate, though not to zero [3]. However, the administration of prophylactic antibiotics 30 to 60 minutes before surgery, decreases the incidence of SSI [4-10]. Obviously, remote infections increase SSI. Other contributing factors are the type of surgery and secondary infections. The object of this study was to assess SSI, and the incedence of the factors contributing to such infections at Imam Khomeini Hospital in Iran.

## Methods

In this study, 802 patients in a teaching hospital during the 15 months from April 2002 to July 2003 underwent abdominal surgery and were studied for SSI and the factors affecting it. Initially, 884 patients were recruited for the study but 82 were excluded on the basis of the following criteria: deficient medical records; patients operated at a different hospital and subsequently transferred to Imam's Hospital: or patients deceased during the operation or within the following 30 days.

The dependent variable in this study was abdominal surgical site infection, defined as redness, swelling, pain, temperature above 38°C, during the 30 days after operation. The independent variables were: age, sex, site operated, body mass index, time of shaving the site of incision, administration of prophylactic antibiotics, type of surgical operation, duration of operation, duration of preoperative bed stay; preoperative shower, type of shaving, and accompanying conditions.

The data were collected through a 20-item questionnaire. The stages for data collection and information completion were as follows: identification of patients; preoperative interview; postoperative interview; record completion; weekly examinations and telephone follow-ups for 30 days following operation; and pre-discharge examinations. The collected data were analyzed by the SPSS 10 package, using Student's t-test for continuous variables and chi-square test.for categorical variables.

To the best of our ability, this study was conducted with due attention to research ethics.however, problems met in the follow-ups the patient's discharge imposed limitations on the study

## Results

Of the 802 patients studied who had undergone abdominal surgery 139 (17.4%) suffered from SSI as defined in Table 1. No infections were observed in the other 663 cases (82.7%). So far as wound type was concerned, we found clean wounds in 109 cases (13.6%); clean-contaminated wounds in 214 cases (26.7%); contaminated wounds in 307 cases (45.8%); and dirty infected wounds in 112 cases (14%). While 255 cases (28.1%) did not shower before the operation, the other 577 patients (71.9%) did.

**Table 1:** Population Distribution based on the type of operation and SSI Incidence.

**Type of operation**	**Frequency**	**Percent**	**SSI incidence**	**Percent**
splenectomy	24	3	--	--
cholecystectomy	272	33.9	25	9.2
umbilical hernia	35	4.4	5	14.3
appendicitis	132	16.5	19	14.4
stomach cancer	58	7.2	23	39.7
Excision biopsy	15	1.9	--	--
laparotomy	142	17.7	25	17.6
cystectomy	9	1.1	4	44.4
colon cancer	24	3	14	58.3
colostomy	15	1.9	10	66.7
laparoscopy	18	2.2	--	--
abdominal mass	10	1.2	2	20
ileostomy	10	1.2	5	50
Intestinal adhesion	5	0.6	4	80
others	33	4.2	4	12.1
total	802	100	140	17.4

The body mass index for 73 patients (9.1%) was above 30, indicating obesity. Over half the patients (403 cases) suffered from accompanying conditions such as diabetes, high arterial blood pressure, kidney or liver failure, malignancy, febrile condition, cardiac disorders, thyroid disorders, blood disease, chronic obstructive pulmonary disease, convulsion, hyperlipidemia, or immunological disorders; or had previously undergone surgical operations. The rest were free of accompanying conditions (Table 2).

**Table 2:** SSI Distribution based on the factors involved.

**Variable**	**Classification**	**SSI (+)**		**SSI (-)**		**total**		**P. Val.**
			
		**N**	**%**	**N**	**%**	**N**	**%**	
**Age**	**Age groups under 25**	4	3.7	103	96.3	107	100	**<0.001**
	**Age groups 25–65**	102	18.1	462	81.9	564	100	
	**Age groups above 65**	33	25.2	98	74.8	131	100	

**Sex**	**Female**	61	15.1	344	84.9	405	100	**<0.093**
	**male**	78	19.6	319	80.4	397	100	

**Type Of Wounds**	**Clean wounds**	5	4.6	104	95.4	109	100	**<0.001**
	**Clean contaminated wounds**	9	4.2	205	95.8	214	100	
	**Contaminated wounds**	115	31.3	252	68.7	367	100	
	**Dirty – infected wounds**	10	8.9	102	91.1	112	100	

**Type Of Operation**	**Urgent**	29	14.9	166	85.1	195	100	**<0.001**
	**elective**	110	18.1	497	81.9	607	100	

**Operation Duration**	**Below 1.5 hours**	9	5.4	157	94.6	166	100	**<0.001**
	**1.5 – 4 hours**	121	19.5	501	80.5	622	100	
	**Above 4 hours**	9	64.3	5	35.7	14	100	

**preoperative bed stay**	**Emergency**	24	11.7	181	88.3	205	100	**<0.018**
	**1 – 15 days**	100	18.6	439	81.4	539	100	
	**More than 15 days**	15	25.9	43	74.1	58	100	

**Shaving Time**	**One hour before operation**	29	14.5	171	85.5	200	100	**<0.001**
	**12 hours before operation**	110	18.3	492	81.7	602	100	

**showering Before op.**	**Taken**	80	13.9	497	86.1	577	100	**1.00**
	**none**	59	26.2	166	76.8	255	100	

**Body mass index**	**Under 20**	10	17.2	48	82.8	58	100	**0.692**
	**Ranging 20.1 – 25**	73	16	383	84	456	100	
	**Ranging 25.1 – 30**	42	19.5	173	80.5	215	100	
	**Above 30.1**	14	19.2	59	80.8	73	100	

We found no significant correlations between SSI incidence and sex or preoperative shower. However, correlations with duration of operation, duration of preoperative bed stay, electivity of surgery, lengthening of preoperative shaving time, increasing age, wound infection, site of surgery, type of incision, accompanying disorders, and type of prophylactic antibiotic administered before operation were all significant at p < 0.001. Although differences in SSI rates were not significantly related to BMI, a trend was apparent: SSI rate was higher with low and high BMI.

The following prophylactic antibiotics were used: ampicillin, gentamicine, cephalothin, metronidazole, ceftriaxone and cefazoline (table 3).

**Table 3:** SSI Distribution based on the type of prophylactic antibiotic administered.

variables SSI	cefazolin				ceftriaxone				Metronidazole				cephalothin				Gentimicine				Ampicillin			
	A		NA		A		NA		A		NA		A		NA		A		NA		A		NA	

A	N	%	N	%	N	%	N	%	N	%	N	%	N	%	N	%	N	%	N	%	N	%	N	%

positive	42	12.1	97	21.3	73	24.5	66	13.1	79	24	60	12.7	19	17	120	17.4	70	30.4	69	12.1	5	50	134	16.9
negative	305	87.9	358	78.7	225	75.5	438	86.9	250	76	413	87.3	93	83	570	82.6	160	69.6	503	87.9	5	50	658	83.1
total	347	100	455	100	298	100	504	100	329	100	473	100	112	100	690	100	230	100	572	100	10	100	792	100
P-Value	P = 0.001				P < 0.001				P < 0.001				P = 1.000				P < 0.001				P = 0.018			

A = applied

NA = not applied

## Discussion

For the 802 participants in this study, the SSI reported was 17.4%, which is well above the 14–16% reported in other studies [1]. There were particularly high values in cases of umbilical hernia (14.3% compared with previously reported 5%) and stomach cancer (39.7%; in previous studies 20%) [4]. Increasing age is correlated with greater likelihood of certain chronic conditions, malnutrition and a fall in the body immunological efficiency, causing more extensive SSI [4]. The present findings supported this argument (p = 0.001).

SSI is not correlated with sex [5], in agreement with previous findings (p = 0.093). The literature shows that SSI increases with obesity, one reason being a decrease in blood circulation in fat tissues [7]. Malnutrition is another factor predisposing to SSI [5]. In this study we considered a BMI of above 30 obese and that of below 20 as malnutrition, found no significant correlations between the two ranges and SSI extensity (p = 0.692). But, the previously reported correlation between SSI and pre-operation bed stay (p = 0.018) [4,5] was supported by this study. This is one of the factors to be taken into account. Thus by reducing pre-operation bed stay we may decrease SSI. The findings of this study also proved the risk of SSI to be less in elective surgeries than those referred to emergency departments as cases of acute abdomen, which could result from lack of readiness for operation on the patient's side. Here we should reduce risk factors by preparing the patient for the urgent operation as much as possible.

The findings supported the literature by showing that administration of prophylactic antibiotic half an hour before the operation would bring about the best results and the lowest SSI [10]. This was proved for all antibiotics (p = 0.001) with the exception of cephalothin with (p = 1), which requires a lot more research.

The literature shows that with the duration of above 2 hours, the risk of SSI increases [4]. The average time in this study was 2.24 hours, which must be reduced to below 2 hours although the nature of surgical operations is not always the same.

The time of shaving when it approaches the operation and if done by clippers, reduces the SSI risk. In this study the two times: one hour before surgical operations and 12 hours before that were contrasted which supported previous findings with p = 0.001. This is one area where we can lower the risk by approximating the time of shaving as much as possible to that of operation.

Other such factors quoted in the literature as the conditions of the operating theatre, personal hygiene, acompanying diseases, immunological disorders, smoking, techniques of surgery, the surgeon's expertise, duration of surgical scrub, preoperative skin preparation, poor hemostasis, failure to obliterate dead space, tissue trauma, and inadequate sterilization of instruments, which were not included in this study might be considered as confounding factors.

## Conclusion

Considering the relatively higher rate of SSI in this study (17.4% compared with the 14% quoted in the literature), especially in such cases as stomach cancers and umbilical hernia, where the rate is considerablly higher, we should carefully reconsider the whole operation procedure. In general, we should do our best to reduce the average operation duration to less than 2 hours and the average preoperative bed stay to less than 4 days. Thus, the present average of 11.2 days for the total bed stay would be reduced to less than 11 days. The time of shaving should approximate the operation time as much as possible. Finally, when the administration of prophylactic antibiotics is required, cefazoline is recommended to substitute cephalothin.

Razavy: main researcher (design, analysis, report)

Ibrahimpour: (implementation, analysis)

Sabouri Kashani: (report, edit)

Jafarian: (counsult)

## Competing interests

The author(s) declare that they have no competing interests.

## Pre-publication history

The pre-publication history for this paper can be accessed here:


